# Local excision in mid-to-low rectal cancer patients who revealed clinically total or near-total regression after preoperative chemoradiotherapy; a proposed trial

**DOI:** 10.1186/s12885-019-5581-9

**Published:** 2019-04-29

**Authors:** Jong Lyul Lee, Seok-Byung Lim, Chang Sik Yu, In Ja Park, Yong Sik Yoon, Chan Wook Kim, Seong Ho Park, Jong Seok Lee, Yong Sang Hong, Sun Young Kim, Jeong Eun Kim, Jong Hoon Kim, Jin-hong Park, Jihun Kim, Minkyu Han

**Affiliations:** 1 Division of Colon and Rectal Surgery, Department of Surgery, University of Ulsan College of Medicine, Asan Medical Center, 88, Olympic-ro 43-gil, Songpa-gu, Seoul, 05505 Korea; 2 Department of Radiology, University of Ulsan College of Medicine, Asan Medical Center, 88, Olympic-ro 43-gil, Songpa-gu, Seoul, 05505 Korea; 3 Department of Medical Oncology, University of Ulsan College of Medicine, Asan Medical Center, 88, Olympic-ro 43-gil, Songpa-gu, Seoul, 05505 Korea; 4 Department of Radiation Oncology, University of Ulsan College of Medicine, Asan Medical Center, 88, Olympic-ro 43-gil, Songpa-gu, Seoul, 05505 Korea; 5 Department of Pathology, University of Ulsan College of Medicine, Asan Medical Center, 88, Olympic-ro 43-gil, Songpa-gu, Seoul, 05505 Korea; 6 Department of Clinical Epidemiology and Biostatistics, University of Ulsan College of Medicine, Asan Medical Center, 88, Olympic-ro 43-gil, Songpa-gu, Seoul, 05505 Korea

**Keywords:** Rectal cancer, Preoperative chemoradiotherapy, Regression, Postoperative complication, Local excision, Total mesorectal excision

## Abstract

**Background:**

Preoperative chemoradiotherapy (pre-CRT) followed by total mesorectal excision (TME) is currently a standard therapy for locally advanced mid-to-low rectal cancer. Less aggressive, organ-preserving option such as local excision (LE) or watchful wait can alternatively be used for patients who respond well to pre-CRT. High-resolution rectal magnetic resonance imaging (MRI) is one of the most useful methods to assess pre-CRT response, and the MERCURY group has shown that the MR tumor regression grade (mrTRG) correlated with the pathologic TRG. The aim of this study is to compare postoperative complication and oncologic outcomes between LE and TME in mid-to-low rectal cancer patients whose tumors are mrTRG grade 1 (radiological complete remission) or 2 (predominant fibrosis; near-complete remission) after pre-CRT.

**Methods:**

A prospective, double-arm, randomized, open-labeled, single center, clinical trial will be conducted in patients with mid-to-low rectal cancer whose tumors are mrTRG 1/2 after pre-CRT at the Asan Medical Center, Seoul, Korea, after approval from the Institution Review Board. Patient medical records will be de-identified using a serial number to protect personal information. Inclusion criteria will include rectal adenocarcinoma with an inferior border < 8 cm from the anal verge, mrTRG 1/2, age > 20, and provision of informed consent. Postoperative complications will be assessed by Clavien-Dindo Classification Grade. Oncologic and functional outcomes will be collected and risk factors related to these outcomes will be investigated.

**Discussion:**

We believed that the rate of postoperative complication of LE will be comparable to that of TME in mid-to-low advanced rectal cancer patients with a favorable response after pre-CRT.

**Trial registration:**

KCT0002579 (https://cris.nih.go.kr) Dec-2017.

## Background

In the treatment of rectal cancer, total mesorectal excision (TME) improves oncologic outcomes and adding preoperative chemo-radiotherapy (pre-CRT) to TME has been shown to effectively improve local control [1–4]. For this reason, pre-CRT followed by TME is recommended for patients with locally advanced mid-to-low rectal cancer [5–9]. However, significant complications are associated with TME, including anastomotic leak, sexual and urinary dysfunction, and frequent stool passage, on top of the peri-operative morbidity associated with all major surgery [10–12]. In addition, abdominoperineal resection (APR) involves a permanent stoma and low anterior resection (LAR) often involves at least a temporary stoma [8, 12, 13]. The decision of a surgical strategy must take oncologic outcomes, postoperative complications, and quality of life into account.

For patients with favorable responses to pre-CRT, organ-preserving strategies such as local excision (LE) and watchful waiting are becoming more accepted [14–19]. There has been some debate regarding the oncologic safety of LE following pre-CRT in advanced mid-to-low rectal cancer [14–18]. LE following pre-CRT for advanced rectal cancer could be a particularly good option for patients showing a complete pathologic response and achieving tumor regression not only in the primary tumor but also in the mesorectal metastatic lymph nodes [20–24]. Although the tumor regression grade (TRG) obtained using high-resolution rectal magnetic resonance imaging (mrTRG), as proposed by the MERCURY study group, correlated with the pathologic TRG [20, 21, 23, 24], mrTRG did not reflect the pathologic T status. A recent study from our institution showed that a status of mrTRG1 predicted a pathologic complete remission (pCR) with 60.6% accuracy and the projected completion TME rate of mrTRG1 was 25.6%. This results suggests that LE, compared with watchful wait, is more likely to result in pCR [25].

There have been several reports on the feasibility of performing LE following pre-CRT, most of which are focused on either clinical T2–3 rectal cancer or on cases of pCR [26–28]. However, the oncologic safety of LE in patients with a pathologic early T status after pre-CRT has not been established [29]. Therefore, data on long term outcomes with respect to the pathologic status and the type of surgery in patients with locally advanced rectal cancer is needed.

## Methods

### Objective

The purpose of the current study is to determine whether LE in mid-to-low rectal cancer patients with mrTRG 1 or 2 tumors after pre-CRT is safe and effective in terms of surgical and oncologic outcomes.

### Primary outcome measures

All complications within 30 days of the surgical intervention will be assessed using the Clavien-Dindo Classification Grade.

### Secondary outcomes measures

Secondary outcomes will be as follows:

1. Oncologic outcomes: 3-year disease-free survival (% DFS), 3-year overall survival (% OS), and recurrence rate (%).
2. Functional outcomes: quality of life, as measured using EORTC QLQ-C30 (version 3) and rectal function, as measured using manometry, low anterior resection syndrome (LARS) score, and the Wexner Fecal Incontinence Score.
3. The rate of permanent stoma formation (%).
4. Factors affecting complete remission and the accuracy of mrTRG.
5. Oncologic outcome in patient subgroups.

### Study design

**Fig. 1:**
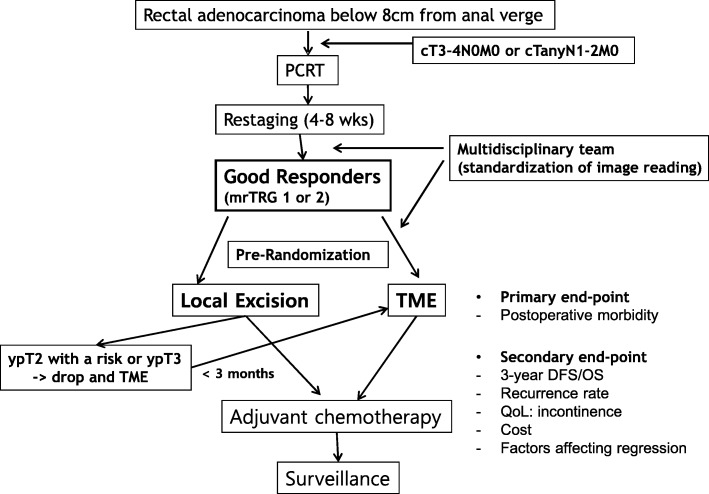
Study design including primary and secondary study endpoints

A prospective, double-arm, randomized, open-labeled, single center, clinical trial was initiated at Asan Medical Center, Seoul, Korea beginning in July 2017. (Fig. 1).

### Study population

Patients who were diagnosed with pathologically confirmed rectal adenocarcinoma < 8 cm from the anal verge with clinically T3 or N+ before pre-CRT and an mrTRG grade 1 or 2 based on post-CRT images will be eligible for the study when they meet the following inclusion criteria:

- Male and female patients between the age of 20 and 80;
- Good performance status (ECOG performance status < 2);
- Capable of providing informed consent.

### Exclusion criteria

- Rectal cancer > 8 cm from the anal verge;
- Microperforated lesion before pre-CRT;
- Concurrent metastasis at a diagnosis;
- Synchronous colorectal cancer;
- Previous history of radiotherapy;
- Other synchronous or metachronous malignancies diagnosed within 5 years of the diagnosis of rectal cancer;
- Enrollment in other clinical trials except for those of adjuvant therapies;
- Pregnant or breastfeeding women.

### Pre-registration procedures

#### Preoperative chemoradiotherapy

Pre-CRT was delivered at 50.4 Gy to the primary tumor and the pelvis in 25 fractions. Concurrent chemotherapy during radiotherapy consisted of 5-fluorouracil (5-FU; 375 mg/m^2^/day) and leucovorin (LV; 20 mg/m^2^/day) or capecitabine (825 mg/m^2^). 5-FU and LV were given on three consecutive days for two courses during the first and fifth weeks of radiotherapy, while capecitabine was given twice daily during the entire radiotherapy period without a weekend break.

#### Preoperative restaging

For assessing the pre-CRT response, digital rectal examination, sigmoidoscopy, abdomino-pelvic computed tomography (CT), chest CT, trans-rectal ultrasonography and MRI were performed within 2 weeks before surgery. Two board-certified abdominal radiologists independently assessed the response to CRT on post-CRT MRI using mrTRG (1–5) according to published definitions: 1, complete radiologic response; 2, dense fibrosis with no obvious residual tumor; 3, > 50% fibrosis or mucin with visible residual tumor; 4, small areas of fibrosis or mucin but mostly tumor, or tumor growth; 5, same appearance as baseline. The enrollment of a study cohort requires a multidisciplinary team including a colorectal surgeon, medical oncologist, radiation oncologist, and radiologist.

### Treatment strategies

#### Standard care of the control arm

TME is the standard treatment and is undertaken 6–8 weeks after the completion of pre-CRT. All operations were performed by experienced colorectal surgeons (> 100 rectal cancer cases per year). Temporary diverting ileostomy is occasionally performed in cases of a very low anastomotic level with considerable tension, air leak, or severe comorbidities. We do not perform lateral lymph node dissection routinely.

#### Investigational treatment of the experimental arm

LE will be performed via conventional transanal excision or transanal minimal invasive surgery. During LE, remnant scar tissue will be excised with at least a 1 cm resection margin. Patients who have poorly differentiated or mucinous ypT2 tumors with lympho-vascular invasion, perineural invasion, tumor budding, and close resection margins (< 1 mm) will be recommend for TME within 3 months. If the patient undergoes TME, he or she will be evaluated as part of the TME group. If the patient does not choose to undergo TME, he or she will be excluded from the study and followed-up for additional subgroup analysis.

#### Postoperative follow-up schedule

Patients will be followed for 3-year after surgery. Follow-up will consist of a physical examination and blood tests, including carcinoembryonic antigen (CEA) levels every 6 months after surgery. Chest X-ray and abdomino-pelvic CT every 6 months after surgery, chest CT will be performed every 6–12 months, and colonoscopy performed at 6–12 months and 24–36 months after surgery (Table 1).

**Table 1:** Follow-up schedule and study procedure for study cohort

Documentation	Restaging	LE vs. TME	Visit 1	Visit 2–6
Study procedures	Screening	Surgery	Checking complications	Routine checkup
Period after pre-CRT	4–8 weeks	4–8 weeks	8–16 weeks	Every 6 months after Op.
Getting permission	●			
Screening	●			
Assigning serial number	●			
Demographics	●			
Physical exam	●		●	●
Heart/ lung evaluation	●			
Chest CT	●			● (Selective)
APCT	●			●
MR, rectal cancer	●			● (Selective)
Endoscopy	●			● (Selective)
Laboratory test	●		●	●
Endorectal sonography	●			
Manometry/questionnaire	●			● (Selective)
Checking complications			●	●
Selection of surgery type		●		

*Pre-CRT* Preoperative chemoradiotherapy, *Op*., Operation, *LE* Local excision, *TME* Total mesorectal excision, *CT* Computed tomography, *APCT* Abdomino-pelvic CT, *MR* Magnetic resonance

#### Primary outcome

The primary endpoint of the current study is the rate of postoperative complications. Postoperative events occurring up to 30 days after surgery will be evaluated by the Clavien-Dindo Classification, any events grade 2 or higher will be considered postoperative complications.

#### Secondary study endpoints

1. Disease-free survival rate (%), overall survival rate (%) and recurrence rate (%) at 3 years.

2. Functional outcomes, including quality of life (EORTC-E030) and score of fecal incontinence (Wexner’s score) at 6, 12 and 24 months after surgery.

3. Rate of permanent stoma formation (%) at 3 years.

4. Accuracy of mrTRG (positive predictive value and negative predictive value).

#### Sample size calculation

The sample size was calculated based on two-sided non-inferiority test with for 80% power and a significant level of 0.05, one-sided. We assume that the rate of postoperative complications on day 30 will be 15% in the TME group and 15% in the LE group, with a non-inferiority margin of 15%. Based on these calculations, a total of 67 patients should be allocated to each group; assuming a 5% dropout rate, we plan to enroll 140 patients. The 15% rate of postoperative complications cited above is based on published findings [26–28, 30].

#### Randomization and data management

After checking the response of pre-CRT, the participants with mrTRG 1 or 2 tumor will be assigned using random-number table with sealed envelopes by an external research worker who control the data. According to mrTRG 1 or 2, the allocation sequence will be stratified. The external research worker will collect the data and a statistician who provided the sample size calculation and data analysis plan will be analyze the data. The data entry, coding, security and storage with backup will be processed by the external research worker and simultaneously the statistician will check the accuracy and the value of the data. After finishing the collection of data, the external research worker, the statistician will access the dataset and investigators will access after analyses of the dataset.

### Data analysis

The intent-to-treat (ITT) analysis set will include all subjects who are randomized into a surgery group. The modified ITT analysis set will include all subjects who undergo pre-CRT. The per protocol (PP) analysis set will include only the subjects in the ITT group who complete the study protocol. Patients who withdraw informed consent or who develop serious adverse events will be excluded from the PP analysis set. The modified ITT set will be used for analysis of primary and secondary study endpoints. The PP analysis set will be used for subgroup analysis. Baseline characteristics will also be analyzed in the ITT group.

Variables and outcomes will be analyzed using chi-square tests (for proportions) or Fischer’s exact test, as appropriate. Continuous data will be analyzed using Student’s t-test or Mann-Whitney U tests, as appropriate. Cumulative risk rates will be calculated using the Kaplan-Meier method and compared using log-rank tests. Multivariate analyses using binary logistic regression will be used to assess the risk of postoperative complications. Statistical significance will be defined as *p* < 0.05, and all analyses will be performed using SPSS software, version 22 (IBM Corporation, Armonk, NY, USA).

### Safety

The main outcome will be the detection and documentation of postoperative complications. The severity of complications will be classified according to the Clavien-Dindo Classification Grade and will be registered in the case reporting form. Currently, the oncologic outcome of pre-CRT followed by LE is considered to be equivalent to that of pre-CRT followed by TME for patients who respond well to pre-CRT. If the pathologic results show ypT2 with risks, ypT3 or ypT4, surgeons will recommend to perform TME for oncologic safety of the participants. The current study will meet with all local legal requirements and meet all requirements of the ICH Guideline for Clinical Safety Data Management, Definition and Standards for Expedited Reporting, Topic E2A.

## Discussion

Although pre-CRT followed by TME has become the standard treatment for patients with locally advanced rectal cancer, recent evidences suggests that organ-preserving strategies such as LE and watchful waiting can be used as an alternatively to TME in patients with a favorable response to pre-CRT without compromising oncologic outcomes and high rate of major LARS [14–19, 31]. Numerous tools can be used to assess the tumor response after pre-CRT, including physical examination, endorectal ultrasound, endoscopy, and MRI. MR staging is currently considered one of the most useful methods of evaluating the response to pre-CRT [20, 24, 25]. However, the accuracy rate of mrTRG for predicting pCR is only approaximately 60% [25]. Considering that ypT status reflects the ypStage and that patients with pCR or ypT1N0M0 have a more favorable prognosis [29], accurate examination of tumor status is needed.

Recently, our institution reported a retrospective analysis demonstrating that oncologic outcomes of pre-CRT followed by LE were comparable to those of TME, even in patients with lymph node involvement [27]. A recent meta-analysis also suggested that there are no differences in local recurrence rates, disease-free survival, and overall survival between rectal cancer patients that received LE or TME [28]. In patients with early-stage rectal cancer, several studies found that LE did not compromise the oncologic outcome but did result in organ preservation [28]. Based on previous studies, if lymph nodes are positive after pre-CRT, patients are optimally treated with TME with regional lymph node clearance, in line with the current standard of treatment [29], but a patient that achieves a complete or near-complete response after pre-CRT can consider LE.

In terms of postoperative complications, only one patient (2.1%) in our previous study developed a postoperative complication (perianal fistula) that required prolonged hospitalization [27]. In a recent prospective study of LE in patients with T2 rectal cancer, surgery-related adverse events above grade 3 occurred in 16 patients (21%) and grade 1 or 2 complications occurred in 50 patients (65%) [26]. Although complications of LE after pre-CRT are generally less severe than complications of TME, the rate of overall complications including dehiscence of the rectal suture line resulted from anal pain are higher than TME after pre-CRT [26]. Based on the assumption that the oncologic outcomes of LE and TME are similar for patients with a favorable response (mrTRG 1 or 2) after pre-CRT, further prospective studies on surgical outcomes in LE after pre-CRT are needed.

Pre-CRT followed by LE causes minimal loss of anorectal function and quality of life, similar to watchful waiting [26]. TME results in postoperative loss of anorectal, urinary and sexual function, and also carries the risk of permanent stoma formation [10]. In mid-to-low advanced rectal cancer, the use of pre-CRT has decreased the rate of permanent stoma formation; however, in our recent study, approximately 30% of patients receiving TME after pre-CRT did develop a permanent stoma [27]. A recent prospective study suggested that pre-CRT followed by LE might be an alternative to TME for patients with rectal cancer who have a favorable response to pre-CRT or who seek organ preservation because the Fecal Incontinence Severity Index (FISI) score and the Functional Assessment of Cancer Therapy-Colorectal (FACT-C) scores did not substantially decrease after surgery [26].

Previous studies, even prospective studies for LE or watchful waiting, suffer from the lack of standardized selection criteria. Recently, the MECURY group convened multidisciplinary teams (MDT) to decide which patients should defer surgery and which patients should receive standard treatemtn after pre-CRT [22]. Our institution also reported that case discussions in MDT meetings resulted in altered clinical decisions in > 10% cases for advanced and recurrent colorectal cancer [32]. Based on this experience, in the current study, specialized faculty, including a colorectal surgeon, radiologist, medical oncologist, radiation oncologist, gastroenterologist, and pathologist, will select the study patients that show a favorable response after pre-CRT. The data on postoperative complications in LE and TME after pre-CRT collected through the current study will provide valuable information about the risk factors of LE after pre-CRT.

This prospective study has some weaknesses because this study will be performed based on the real practice in our institution. Recent studies insists that the interval between completion of pre-CRT and the assessment of tumor response is increased up to 12 weeks and this longer interval might increase the rate of pathologic CR [33, 34]. On the other hand, in this prospective study, TME is undertaken 6–8 weeks after the completion of pre-CRT according to the real practice in our institution. The schedule of restaging is designed within 2 weeks before surgery considering waiting time of MRI in our institution and minimizing dropouts, however, almost patients have restaged at 2 or 3 days before surgery on the real practice. About 10% of lymph node infiltration and the 15% of local recurrence in the ypT2 were worrisome for this study [27, 29, 35]. Nevertheless, recent reports showed the overall survival was not significantly different between the LE and TME groups [27, 36, 37]. The evidences of LE for ypT2 tumors are still lack, therefore, our study determines the effect of LE in ypT2 tumors on oncologic outcomes.

## Abbreviations

APR: Abdominoperineal resection
CEA: Carcinoembryonic antigen
CT: Computed tomography
DFS: Disease-free survival
ITT: Intent-to-treat
LE: Local excision
MDT: Multidisciplinary team
MRI: Magnetic resonance imaging
mrTRG: MRI_tumor regression grade
mrTRG1: Radiologically complete remission
mrTRG2: Essentially compete fibrosis
OS: Overall survival
pCR: Pathologic complete remission
PP: Per protocol
Pre-CRT: Preoperative chemoradiotherapy
TME: Total mesorectal excision
TRG: Tumor regression grade
WHO: World Health Organization
